# An Overview of Glutaminyl Cyclase as a Promising Drug Target for Alzheimer’s Disease

**DOI:** 10.3390/biomedicines13061467

**Published:** 2025-06-13

**Authors:** Rasajna Madhusudhana, Emily Boyle, Yana Cen

**Affiliations:** 1 Department of Medicinal Chemistry, Virginia Commonwealth University, Richmond, VA 23219, USA; madhusudhanr@vcu.edu (R.M.); boyleel2@vcu.edu (E.B.); 2 Center for Drug Discovery, Virginia Commonwealth University, Richmond, VA 23219, USA

**Keywords:** Alzheimer’s disease, glutaminyl cyclase, small-molecule inhibitors

## Abstract

Alzheimer’s disease (AD) has become an increasingly pressing concern for the aging population. Current AD treatments mainly focus on cognitive and neuropsychiatric symptoms—with few FDA-approved treatments targeting disease progression itself. The amyloid cascade hypothesis describes the formation and accumulation of β-amyloid (Aβ) oligomers and plaques as a primary event in AD pathogenesis. This hypothesis has served as the foundation of disease-modifying treatment development over the last decade. Recently, glutaminyl cyclase (QC) has been identified as a potential drug target in the amyloid cascade. QC catalyzes the cyclization of Aβ to form pyroglutamated Aβ (pEAβ). pEAβ acts as the seed for the formation of Aβ plaques, thus preventing the formation of pEAβ via QC inhibition, and offers a promising therapeutic strategy against AD. Here, we offer an overview of the pathway QCI research has followed—from the initial testing of imidazole-based inhibitor scaffolds to QCI structural optimization via pharmacophore identification, Varoglutamstat entering clinical trials, and further avenues of bettering specificity and potency for future QCI development.

## 1. Introduction

Alzheimer’s disease (AD) is a progressive neurodegenerative disease and is the most common cause of dementia in the elderly [1]. About 6.7 million Americans were living with AD in 2023 but, as the population ages, this number is projected to nearly double by 2050 [2]. AD is characterized by neuronal loss in the hippocampal region of the brain, extracellular amyloid plaques, fibrillary aggregates of hyperphosphorylated tau proteins, and neuroinflammation [3]. Other clinical symptoms of AD include cognitive impairment, behavioral deficits, and neuropsychiatric symptoms like depression, anxiety, and psychosis [4].

Most of the drugs approved for the treatment of AD are for the management of cognitive and neuropsychiatric symptoms. There are only three FDA-approved disease-modifying treatments (DMTs), including one that was approved as recently as July 2024. All three are monoclonal antibodies that target the amyloid plaques formed in the disease state [5]. Although they are promising therapeutic options, some have a high number of adverse events and there is insufficient credence in the clinical trials regarding their efficacy [6,7]. The pursuit of better AD therapies is in progress, with about 20 drugs currently in phase 3 clinical trials targeting various aspects of AD pathology including the amyloid cascade, the modulation of neurotransmitter receptors, synaptic plasticity, proteostasis, tau tangles, inflammation, metabolism, and circadian rhythm [5].

Neurodegenerative diseases (NDs) such as AD, Parkinson’s disease (PD), dementia with Lewy bodies, multiple system atrophy, and various a-synucleinopathies have very different clinical manifestations but there is one common underlying pathological mechanism that makes them remarkably similar. The progressive accumulation of heterogeneous aggregates of peptides or misfolded proteins, eventually leading to cellular dysfunction and neuronal death in various parts of the brain, is a common hallmark across multiple NDs [8]. This commonality makes this protein aggregation cascade a desirable drug target across different NDs [9]. Even in AD, the beta-amyloid cascade continues to be a popular drug target for the development of DMTs, being the target of one-third of the DMTs in phase 3 trials as of January 2024 [5].

The amyloid hypothesis describes the deposition of β-amyloid (Aβ) oligomers and plaques as one of the primary events in AD pathogenesis [10]. Biomarker studies have also shown that extracellular Aβ deposits accumulate decades before the appearance of mild cognitive impairment in patients [11]. Aβ aggregation and the formation of tau tangles constitute the biochemical phase of AD, followed by the cellular phase, characterized by neuroinflammation and immune responses mediated by microglia and astrocytes. These changes culminate in hippocampal shrinkage, MRI changes, dementia, and cognitive impairment in the clinical phase [12].

Halting or slowing down the biochemical phase of AD by inhibiting the formation of Aβ oligomers and aggregates is a promising therapeutic strategy in theory [13]. In actuality, repeated failures in this direction have compelled deeper investigation of Aβ plaque composition and the mechanism of its formation—revealing both pyroglutamated Aβ (pEAβ) and glutaminyl cyclase (QC), the enzyme responsible for the formation of pEAβ, as potential drug targets. pEAβ is closely associated with synaptic dysfunction, neuronal loss, and chronic neuroinflammation [14,15], partly due to QC’s involvement in chemokine maturation, which promotes microglial activation [16]. Given its central role in generating toxic Aβ species and modulating inflammatory pathways, QC has emerged as a promising therapeutic target in AD. The following sections will discuss the role of QC in AD pathophysiology, its mechanism of action and structural features, and the development of generations of QC inhibitors.

## 2. Pyroglutamated Aβ and Glutaminyl Cyclase in Amyloid Cascade

**Figure 1:**
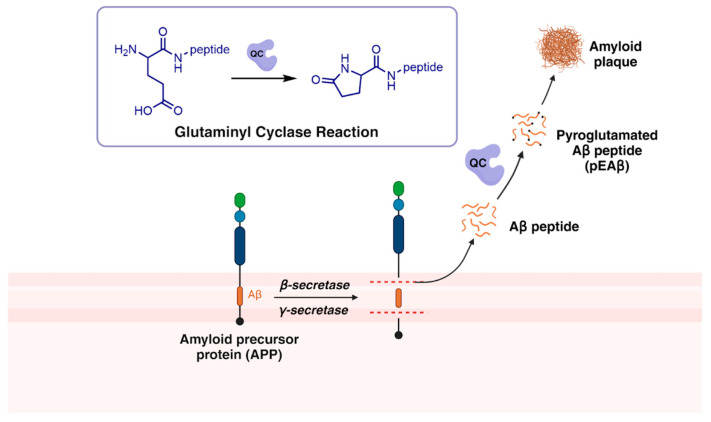
QC in Amyloid Cascade.

Aβ oligomers and plaques are highly heterogeneous. They are composed of peptides of various lengths resulting from the metabolism of amyloid precursor protein (APP). APP is cleaved by three types of proteases; namely, α-, β-, and γ-secretases. The dysregulation of the activities of these proteases gives rise to amyloidosis [17]. Once Aβ peptides are formed, they are further embellished with various posttranslational modifications (PTMs), one of which is pyroglutamation by QCs. QC catalyzes the cyclization of *N*-terminal glutamate residues at positions 3 and 11 to form pEAβ (Figure 1) [18]. pEAβ is more hydrophobic, more stable to *N*-terminal peptidases, has a higher aggregation propensity, and is more neurotoxic compared to Aβ. Cortical pEAβ concentration also shows a higher correlation with cognitive impairment and appears to be more specifically linked to disease progression. pEAβ forms the nucleus of the Aβ aggregates and accelerates their formation in a manner similar to prions [14,15].

QCs’ mRNA levels and protein expression in AD brains, peripheral blood, and cerebrospinal fluid (CSF) were higher compared to age-matched controls [18,19]. QC knock-out in AD mice models resulted in reduced pEAβ load, decreased plaque pathology, and a rescue of the behavioral phenotype; validating that the pharmacological inhibition of QC is a promising therapeutic strategy in early-stage AD [20].

## 3. QC—Structure and Function

QC is a metalloenzyme, with a zinc ion in its catalytic site, that catalyzes a posttranslational cyclization of *N*-terminal glutamate or glutamine residues to form pyroglutamate or pyroglutamine, respectively, in peptides, proteins, or chemokines [21]. Its mechanism is similar to exopeptidases and involves the removal of a water or ammonia molecule. This pyroglutamation is essential for stabilizing various bioactive peptides, chemokines, and hormones. On the other hand, the pyroglutamation of harmful peptides such as Aβ, α-synuclein, and pro-inflammatory chemokines can lead to various disease states [22,23].

There are two isoforms of QC found in humans, secretory QC (sQC) and Golgi-resident QC (gQC) [16]. Both isoforms are ubiquitously expressed throughout the human body. While gQC shows relatively uniform expression across tissues, sQC is more highly expressed in neuronal tissues [24], making it particularly relevant to AD pathology. In the AD brain, sQC expression is further upregulated [25,26]. Additionally, sQC exhibits higher enzymatic activity on synthetic substrates compared to gQC [24], likely due to differences in their active sites, which may influence substrate binding and catalysis.

**Figure 2:**
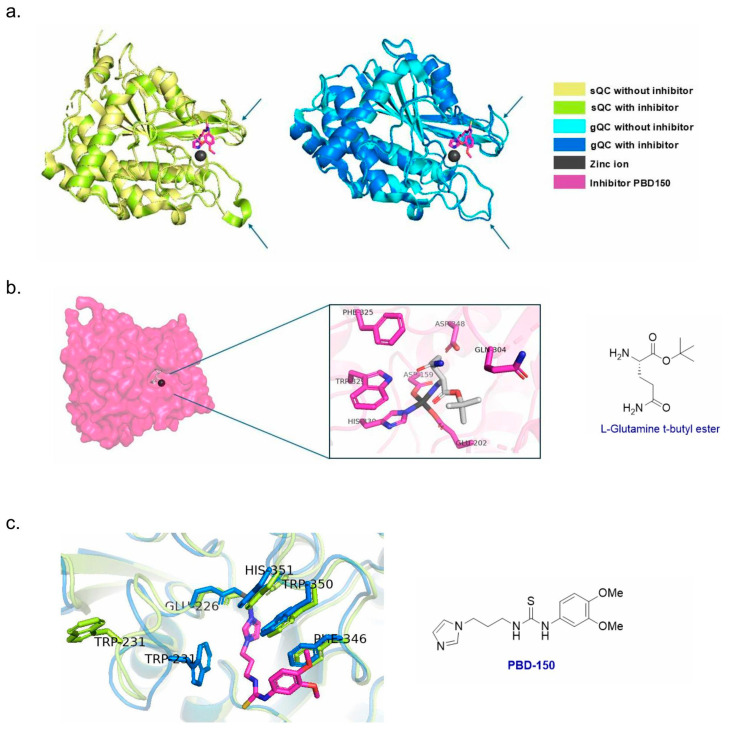
Structures of QCs. (**a**) Structures of gQC and sQC with and without an inhibitor. The arrows indicate loop regions near the active site, which appear more flexible in gQC; (**b**) the binding of the L-glutamine t-butyl ester to sQC, highlighting key interactions; (**c**) the superimposed structures of sQC and gQC bound to **PBD-150**, showing a conformational difference in the indole side chain of W231.

The structures of both isoforms contain many similarities, featuring a common globular fold with a mixed α/β architecture (mimicking an open sandwich topology, Figure 2a). Additionally, the proteins also contain 3_10_-helices and unstructured loops. Both structures include a Zn^2+^ ion located in the active site, coordinated by three conserved amino acids (AAs) including Asp, Glu, and His (D159, E202, and H330 for sQC; D186, E226, and H351 for gQC) [16]. There are also notable structural differences between the two isoforms, including *N*-terminal signaling sequences which are responsible for either secretion or anchoring at the Golgi, and the size of the active sites. Compared to sQC, gQC harbors a relatively wider and more open active site due to the conformational flexibility of several loops (Figure 2a) [16,27]. In addition to the highly conserved catalytic AAs described above, Q304, D248, and W329 were also found to interact with the substrate glutamine t-butyl ester in sQC (Figure 2b) [27]. Upon superimposing the co-crystal structures of both QC isoforms bound to the inhibitor **PBD150**, the active site residues appeared largely conserved; however, a notable difference was observed in the conformation of W321 [28]. In gQC, the indole side chain of W321 was oriented away from the active site (Figure 2c). This positional shift was proposed to contribute to the reduced enzymatic activity of gQC.

## 4. QC Inhibitors

### 4.1. The Prototype

**Figure 3:**
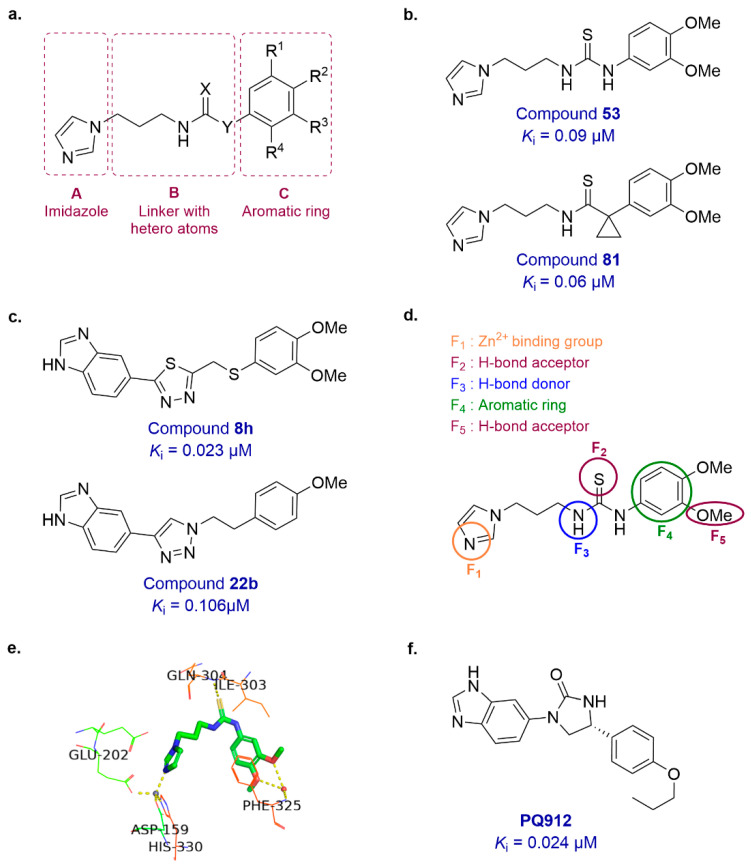
QC inhibitors. (**a**) Prototypes of QCIs showing A, B, and C components; (**b**) imidazole-containing QCIs; (**c**) benzimidazole-containing QCIs discovered by pharmacophore-based virtual screening; (**d**) the pharmacophore of QCIs superimposed on compound **53;** (**e**) the interaction of compound **53** with the active site residues present in QC; (**f**) the structure of **PQ912**.

Imidazole was found to weakly inhibit QC due to its ability to coordinatively bind to the zinc ion in the active site [29]. Investigation of the substrate specificity of QCs revealed that they favor aromatic side chains at the penultimate position next to the *N*-terminal glutamine/glutamate. The preliminary screening of various substituted imidazoles showed that imidazole attached to an aromatic moiety by a long alkyl spacer was preferred. Based on this understanding, a library of imidazole-containing QCIs was synthesized [29]. The prototypical structure of first-generation QCIs consists of three components: A—the imidazole, B—a heteroatom-containing linker such as thiourea or thioamide, and C—an aromatic component (Figure 3a). Various combinations of these components were screened against QC, out of which compounds 53 (same as **PBD150**) and 81 emerged to be the most potent inhibitors (Figure 3b) [29].

A pharmacophore was then constructed using a flexible alignment of both compounds **53** and **81** with the substrate H-Gln-Phe-Ala-NH_2_. This pharmacophore filter was used to screen a library containing 653,218 preprocessed lead-like compounds. This virtual screening, followed by in vitro testing, yielded two potent QCIs: **8h** and **22b** (Figure 3c) [30]. In these molecules, the A component is a benzimidazole moiety instead of an imidazole and the B component is now a heterocycle, while the C component remains unchanged. The *K*_i_ values of these new QCIs were still comparable to compounds **53** and **81** [30]. The pharmacophore can be easily justified when visualized to be positioned in the catalytic domain of hQC (Figure 3d,e). The F1 of the pharmacophore is essential for coordinating to the zinc ion. F2, F3, and F5 form H-bonds with the peptide backbone. F2 and F5 act as H-bond acceptors whereas F3 acts as a H-bond donor. The aromatic ring F4 forms pi–pi stacking interactions with F325. These interactions have been confirmed by mutagenesis studies as well [30].

### 4.2. PQ912

Based on the insights obtained from the comprehensive drug discovery campaign for QCIs, a new compound **PQ912** was designed and advanced to preclinical development by Probiodrug AG (Figure 3f). **PQ912** showed the competitive inhibition of hQC with a *K*_i_ value of 25 nM [31]. The oral administration of **PQ912** in transgenic AD mice (hAPP_SL_xhQC) at a dose of 0.8g/kg for one week resulted in more than 60% target occupancy in the CSF and brain [31]. The treatment of transgenic AD mice (two different models including hAPP_SL_xhQC and 5xFADxhQC) with the same dose of **PQ912** also reduced pEAβ load in both prophylactic and therapeutic treatment paradigms. The mice’s performance in the Morris water assessment of spatial learning and memory showed a significant improvement after a preventative long-term treatment regime, as well as in the therapeutic short-term treatment regime. Furthermore, there was no difference in the testosterone and thyroxine levels of the experimental mice, indicating that **PQ912** selectively inhibits the interaction of hQC with Aβ and does not affect its other physiological roles [31].

Following the promising preclinical testing, **PQ912** was advanced to clinical testing by Vivoryon Therapeutics under the name Varoglutamstat. Varoglutamstat was generally safe at a dose of 800 mg daily. The treatment group performed slightly better than the placebo group in most of the exploratory endpoints for efficacy such as QC activity, neuronal injury, glial activation biomarkers, and cognitive assessments like the One Back test, although these differences were not statistically significant in most cases [32].

In the Phase 2b study, Varoglutamstat did not meet its primary endpoint and did not show a statistically significant difference in cognition over time. The primary endpoint was measured by a combined score (Z-score) of the Cogstate neuropsychological test battery (NTB), called “Cogstate 3-item scale” [33]. It includes Detection, Identification, and One Back tests and evaluates memory and attention domains. Additionally, there was no improvement in the Instrumental Activities of Daily Living Questionnaire (A-IADL-Q), and electroencephalogram (EEG) assessments after treatment [34]. The available clinical trial evidence of QCI is limited, with **PQ912** being the only one to have advanced to a Phase 2b study. The lack of robust clinical efficacy in cognitive measures highlights the challenges of pursuing QCIs as monotherapies for AD. This underscores the importance of investigating combinatorial approaches in future drug development efforts which will be discussed in the subsequent Section 5, titled “Multi-Pronged Therapeutic Approaches”.

On a related note, Varoglutamstat remains under investigation as a QCI, with current research focusing on its potential in treating diseases characterized by inflammatory or fibrotic components [35]. This shift in focus stems from a significant improvement in kidney function—measured by estimated glomerular filtration rate (eGFR)—observed in the same Phase 2b study. This effect is likely linked to the role of gQC in the maturation of the chemoattractant chemokine ligand CCL2, a key mediator of neuroinflammation and chronic inflammation [36].

### 4.3. New Generations of QCIs

**Figure 4:**
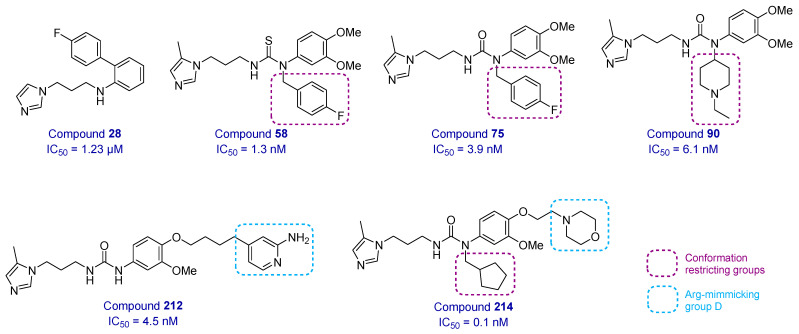
New generations of QCIs with their IC_50_ values for hQC.

Following the advancement of **PQ912** to preclinical studies, more QCIs were developed by modifying existing components or adding new components to the prototypical QCIs to enhance their binding or improve their physicochemical properties [37]. The B component of QCIs, the thiocarbamide linker, was replaced by a diphenyl moiety with an additional phenyl ring for improved blood–brain barrier (BBB) permeability. An imidazole linker was also introduced at the *ortho*-position of the new phenyl ring to generate the new diphenyl conjugated imidazole (DPCI) analogs [38]. Fifteen DPCIs were more potent than the positive control compound **53** (**PD150**). Several of these compounds demonstrated enhanced BBB permeability as confirmed by in silico and in vitro assays. Compound **28** (Figure 4), in particular, showed remarkable inhibitory activities in a dose- and time-dependent manner in APP-transfected HEK293T cells, and also showed improvement in an index of functional deficits in B6C3-Tg AD mice [38].

Docking analysis of prototypical QCIs showed that the *Z*-*E* (bent) orientation of the molecule is preferred over the *Z*-*Z* (straight) orientation. Hence, it was proposed that the addition of a conformation restriction to the nitrogen proximal to the aromatic component would induce the formation of *Z*-*E* conformers. These substitutions improved the potency by 20-fold for thiourea, 100-fold for urea, and 8-fold for amide scaffolds [39]. Among the compounds that were generated, compounds **58** and **75** exhibited low nanomolar IC_50_ values but compound **90** showed the most promising efficacy and drug-like profile (Figure 4). The docking analysis of **90** further confirmed that the *Z*-*E* conformer was indeed the most dominant form with key interactions in the active site [39].

The prototypical QCIs essentially mimic the *N*-terminal Glu-Phe dipeptide region of the natural substrate. Inspired by this, QC inhibitors with an extended scaffold based on the *N*-terminal tripeptide Glu-Phe-Arg of Aβ were investigated. The D region, an additional pharmacophore that mimics the binding interaction of the guanidine moiety of Arg, was identified [40,41]. The incorporation of the D region increased potency by up to 40-fold. Compound **212** (Figure 4), a QCI containing the D region, significantly reduced brain levels of pEAβ and total Aβ in APP/PS1 mice and also restored cognitive function in 5XFAD mice [42]. The incorporation of both conformational restriction and the D region produced highly potent QCIs such as compound **214** with an IC_50_ value of 0.1 nM (Figure 4) [41].

## 5. Multi-Pronged Therapeutic Approaches

### 5.1. Dual Inhibitors of QC and GSK-3β

Glycogen synthase kinase-3 (GSK-3), a proline-directed serine/threonine kinase widely distributed in eukaryotic cells, consists of two isoforms: GSK-3α and GSK-3β [43]. Aberrant GSK-3 activity has been implicated in the development of complex disorders including AD. In AD, GSK-3β is specifically involved in tau hyperphosphorylation and indirectly responsible for Aβ generation [44,45]. Increased GSK-3β activity disrupts the localization and trafficking of β-secretase, leading to excessive Aβ production and aggregation. Additionally, this abnormal GSK-3β activity may trigger brain inflammation and oxidative stress [44,45]. Since both QC and GSK-3β are key targets for AD, the dual inhibition of these enzymes was thought to be a promising strategy.

**Figure 5:**
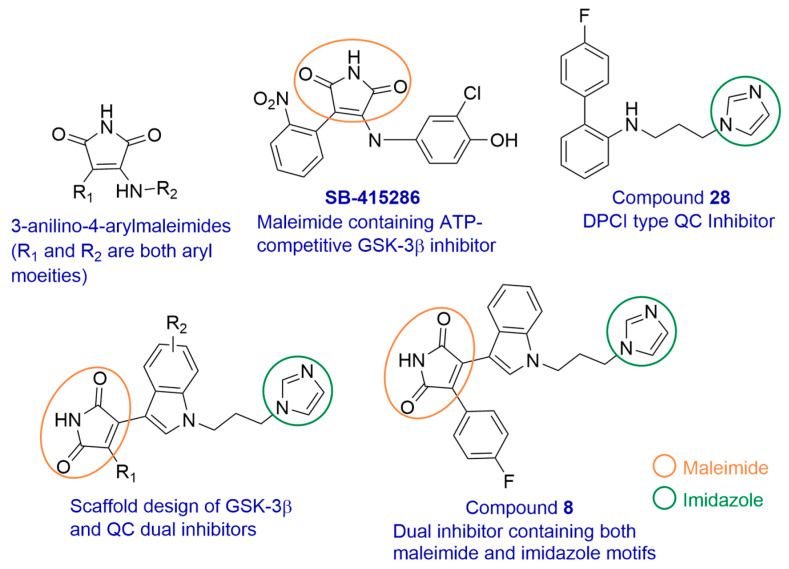
Design of dual inhibitors for QC and GSK-3β.

Reported GSK-3β inhibitors include metal cations, ATP-competitive, and non-ATP-competitive inhibitors [46]. Various small-molecule moieties such as thiazole, indirubin, maleimide, paullone, aloisine, and oxadiazole are known to act as ATP-competitive inhibitors [47]. Maleimide-based compounds are among the most extensively studied GSK-3 inhibitors. Among them, 3-anilino-4-arylmaleimides (Figure 5) represent one of the earliest classes of small-molecule GSK-3 inhibitors. **SB-415286** (Figure 5) is a well-characterized example from this class, identified as a dual GSK-3α and GSK-3β inhibitor [48]. It demonstrated potent inhibition of both isoforms at low nanomolar concentrations and exhibited high selectivity for GSK-3 when tested against a panel of 25 other kinases. Additionally, **SB-415286** showed excellent stability in solution over extended periods while maintaining consistent biological activity [48]. Moreover, **SB-415286** displayed neuroprotective effects in cultured rat cerebellar granule and hippocampal neurons, protecting them from excitotoxicity induced by both NMDA and non-NMDA receptor agonists [49]. Based on these properties, **SB-415286** was selected for the design of dual inhibitors in combination with a DPCI, compound **28** [50]. A series of maleimide–DPCI hybrids were synthesized and evaluated. Among them, compound **8** (Figure 5) significantly reduced the accumulation of both Aβ and pEAβ, decreased hyperphosphorylated tau levels, alleviated cognitive deficits, and reduced anxiety-like behavior in 3×Tg-AD mice [50]. Docking studies revealed that the 4-arylmaleimide moiety binds to the GSK-3β active site, while the imidazole group coordinates with the zinc ion at the QC active site. Additionally, the aryl substituents contributed favorable interactions with both targets [50]. These findings represent the successful design of first-in-class dual inhibitors of QC and GSK-3β, supported by promising in vivo data and offering a strong foundation for further preclinical development.

### 5.2. Combination of QCI with Monoclonal Antibody Therapy

As discussed before, all the currently available FDA-approved therapies for AD are monoclonal antibodies—namely aducanumab, lecanemab, and donanemab [51]. All of these antibodies work towards clearing amyloid oligomers and plaques to decelerate disease progression and alleviate symptoms. However, there remains significant scope to improve the specificity and overall design of current monoclonal antibody therapies. To address this, a combination strategy involving antibody therapy and the QCI Varoglutamstat was envisioned to enhance therapeutic outcomes [52]. PBD-C06 (m6) is a highly specific murine IgG2 antibody that selectively targets pEAβ [53]. It has demonstrated strong binding affinity and avidity for pEAβ oligomers and fibrils, as well as heterogeneous aggregates containing pEAβ peptides. In vitro studies have shown that treatment with m6 inhibited pEAβ fibrillation and prevented pEAβ oligomer-induced cell death [53]. Furthermore, the IgG1 version of m6 significantly reduced cerebral plaque burden and alleviated cognitive impairment in vivo [54]. The combination of m6 and Varoglutamstat was compared to the individual treatments of each component, with doses chosen to achieve approximately a 30% reduction in pEAβ. The Bliss combination indices (CI_Bliss_) were calculated for Aβ fractions showing a significant reduction (via ANOVA). Assuming that the complete inhibition of pEAβ formation is achievable, combination indices between 0.87 and 0.99 were observed. A CI_Bliss_ of 1 indicates an additive effect, suggesting that the combination treatment provides an additive or slightly synergistic effect compared to the single treatments [52]. Although Varoglutamstat alone has not demonstrated significant cognitive benefit in clinical settings, its combination with m6—or other monoclonal antibodies—represents a promising avenue for future investigation.

## 6. Conclusions

Decades of pharmacological studies and drug development efforts are now producing innovative therapeutic options for AD. QCIs are one such promising small-molecule alternative to antibody-based DMTs for AD, but they come with a share of challenges. It is critical to enhance the selectivity of QCIs to inhibit only their reaction with Aβ without hampering its constitutive functions. Another factor to consider is that inhibiting pyroglutamation of Aβ is not enough to effectively halt Aβ plaque formation. The combination of QCIs with anti-Aβ antibodies or using antibody–drug conjugates containing QCIs might be more efficacious treatment strategies as they can overcome some of the aforementioned challenges.

Beyond Alzheimer’s disease, glutaminyl cyclase (QC) has emerged as a therapeutic target in a range of conditions characterized by inflammation and protein aggregation, due to its role in the pyroglutamation and maturation of several pE-modified chemokines and peptide hormones [55]. Notably, **PQ912** (Varoglutamstat) is currently being investigated for the treatment of chronic kidney disease [35]. QCIs have also been extensively studied for their therapeutic potential in thyroid cancer [56]. Recent investigations suggest QCIs can effectively inhibit α-synuclein aggregation thus being a promising therapeutic option for PD, Huntington’s disease, and other α-synucleinopathies [55]. Since there already exists a large number of small-molecule QCIs, they can also be tested in various inflammatory diseases like arthritis or inflammation diseases [57,58]. The limitations remain the same with selectivity being a key issue. Innovative strategies that target the interactions of QC with a specific substrate without hindering the pyroglutamation of essential peptides, hormones, and cytokines are the direction for future research on QCIs.
